# Comparative non-targeted metabolomics reveals distinct metabolic profiles and functional traits in six mung bean (Vigna radiata) varieties

**DOI:** 10.1371/journal.pone.0327962

**Published:** 2025-07-09

**Authors:** Xinting Shen, Huifang Shen, Fei Wang, Yao Wang, Rui Zhao, Zhebin Li, Ye Zhou, Xinmiao Yao

**Affiliations:** 1 Food Processing Research Institute, Heilongjiang Academy of Agricultural Sciences, Harbin, China; 2 Heilongjiang Province Key Laboratory of Food Processing, Harbin, China; 3 Heilongjiang Province Engineering Research Center of Whole Grain Nutritious Food, Harbin, China; University of Kotli, PAKISTAN

## Abstract

This study aimed to investigate the metabolic differences among six mung bean (Vigna radiata) varieties cultivated in Heilongjiang Province, China, using non-targeted metabolomics based on high-resolution mass spectrometry. Principal component analysis (PCA) and orthogonal partial least squares discriminant analysis (OPLS-DA) were employed to explore and distinguish the metabolomic profiles across different varieties. A total of 547 metabolites were identified, including fatty acids (9.69%), phenolic acids (7.86%), amino acids and derivatives (5.12%), among others. PCA revealed that the first two principal components (t[1] and t[2]) accounted for 20.1% and 17.0% of the total variance, respectively, indicating significant differentiation among varieties. Differential metabolite analysis demonstrated that GLD07_03 was enriched in defense-related compounds such as lignans, terpenoid lactones, and methyl salicylate, suggesting enhanced antibacterial and antioxidant capacity. BL13_1 showed higher metabolic activity in glycolysis and the mevalonate pathway, with L-lactic acid and mevalonate levels 57.2% and 33.8% higher than those in GLD07_03. NL2_1 and NL4_1 accumulated high levels of amino acids (e.g., L-aspartic acid, increased by 161%), nucleosides, and flavonoids, contributing to superior antioxidant potential. T1114111_1 exhibited higher levels of fatty acids and alkaloids (e.g., trigonelline, increased by 25.2%), associated with improved membrane stability and antibacterial activity. JL13_1 displayed elevated levels of D-proline and (7Z,10Z)-7,10-hexadecadienoic acid (increased by 26.5% and 34.8%, respectively), suggesting advantages in osmotic regulation and membrane homeostasis. These findings reveal distinct metabolite profiles and biochemical traits among mung bean varieties, providing valuable insights for varietal identification, nutritional evaluation, and breeding for enhanced stress tolerance. The study also offers a scientific basis for the development of functional mung bean products and future breeding strategies focused on metabolic traits.

## 1. Introduction

Mung bean is one of the important edible legumes [1].Currently, more than 6 million hectares of mung beans are cultivated worldwide (about 8.5% of the global legume area) [2].Mung bean, as a traditional multigrain bean with homologous medicine and food, has high nutritional value [3,4]. Although mung beans contain rich nutrients, protein, fat, a variety of trace mineral elements and a variety of vitamins. However, there are also anti-nutritional factors in the seeds to varying degrees, such as phytic acid, protease inhibitors, tannins, etc. Anti-nutritional factors react with minerals, proteins, lipids, carbohydrates and other substances in mung beans to form complexes, thereby reducing nutritional value and affecting the absorption of minerals, proteins and other nutrients by the human body.Compared with other legumes, mung beans have high protein content, moderate starch content, low fat content, less phytic acid production, and higher nutritional utilization value for human beings, and are considered to be an ideal health food for human beings [5]. Studies in recent years have shown that a variety of bioactive substances contained in mung beans have good functions of preventing and controlling diseases and regulating human physiological functions [6,7], and these functions are related to the internal components and metabolic processes of mung beans.

Metabolism is the general term for all biochemical changes in life activities [8], and metabolic activities are the essential characteristics and material basis of life activities [9]. Metabolomics is the quantitative analysis of all metabolites in organisms [10] and the search for the relative relationship between metabolites and physiological and pathological changes. Metabolomics primarily focuses on the systematic identification and quantification of low-molecular-weight metabolites (typically <1000 Da), which serve as substrates and intermediates in various metabolic pathways. These small molecules are involved in key physiological and biochemical processes, such as energy production, cell signaling, and growth regulation, and represent the final downstream products of complex biological activities [11]. In recent years, metabolomics has become a powerful tool in food science, enabling detailed profiling of nutritional compounds, quality evaluation, and authentication of food origin [12]. Among metabolomics strategies, non-targeted metabolomics provides a global overview of the metabolic state without prior selection of compounds, allowing for the discovery of significantly altered metabolites between conditions and their functional interpretation [13–15]. This comprehensive approach not only facilitates biomarker discovery but also sheds light on metabolic pathways and biological functions associated with genotype, phenotype, and environmental responses [16,17].

Metabolic profiles can vary significantly among different varieties of the same crop. A comprehensive comparison of these metabolic differences enables the identification of specific compounds that may confer enhanced nutritional value or health benefits to humans [18,19]. Although mung beans are increasingly recognized for their nutritional and medicinal properties, there remains a lack of systematic studies characterizing the full range of metabolite diversity across different varieties. Most previous research has concentrated on macronutrients or isolated bioactive components, without capturing the broader metabolic landscape that underpins phenotypic diversity. Therefore, the present study employed a non-targeted metabolomics approach to profile and compare the metabolic compositions of six mung bean genotypes cultivated in Heilongjiang Province, China. The primary objective was to identify characteristic metabolite markers associated with key functional traits, including antioxidant capacity, stress resistance, and nutrient enrichment. By uncovering these metabolic distinctions, this study deepens our understanding of mung bean biochemical variability and offers valuable insights for germplasm resource evaluation, functional food development, and precision breeding strategies.

## 2. Materials and methods

### 2.1. Materials and reagents

Six mung bean (Vigna radiata) varieties—JL13−1, GLD07−03, T111411-1, BL13−1, NL2−1, and NL4−1—were selected for this study. These varieties were cultivated under uniform agronomic conditions at the experimental field of the Qiqihar Branch, Heilongjiang Academy of Agricultural Sciences, and were harvested in 2023. Each sample was subjected to three independent biological replicates for metabolite analysis. All metabolomic profiling was conducted using LC-MS/MS-based non-targeted metabolomics.

Methanol, acetonitrile, and acetic acid used in the extraction and analysis were of analytical grade.

### 2.2. Instruments and equipment

The ultra-performance liquid chromatography coupled with high-resolution mass spectrometry (UPLC-Q-Orbitrap HRMS, Vanquish Flex-Q Exactive Plus) was used for data acquisition. A refrigerated centrifuge (Heraeus Fresco 17) was used for sample processing.

### 2.3. Experimental methods

#### 2.3.1. Metabolite extraction.

Mung bean samples were ground using a tissue grinder and passed through a 100-mesh sieve. The extraction procedure was adapted from previously reported methods [20,21]. Briefly, 20 mg of homogenized mung bean powder was mixed with 1000 μL of extraction solvent (methanol:water = 3:1, v/v). The mixture was vortexed and subjected to grinding at 35 Hz for 4 minutes, followed by sonication in an ice-water bath for 5 minutes. This process was repeated two to three times. The samples were then incubated at –40 °C for 1 hour and centrifuged at 12,000 rpm for 15 minutes at 4 °C. The supernatants were collected and transferred into autosampler vials for LC-MS/MS analysis. Quality control (QC) samples were prepared by pooling equal aliquots from each sample.

#### 2.3.2. LC-MS/MS analysis.

Mobile phase A consisted of water containing 0.01% acetic acid, while mobile phase B was a mixture of isopropanol and acetonitrile (1:1, v/v). The autosampler tray temperature was set at 4 °C, and the injection volume was 2 μL. The mass spectrometry parameters were as follows: sheath gas flow rate, 50 Arb; auxiliary gas flow rate, 15 Arb; capillary temperature, 320 °C; full MS resolution, 60,000; MS/MS resolution, 15,000; collision energy (stepped), 20/30/40 eV; spray voltage, + 3.8 kV (positive mode) or –3.4 kV (negative mode). Raw data were collected using UPLC-Q-Orbitrap HRMS and processed with Compound Discoverer 3.3 for peak detection, retention time alignment, peak integration, and metabolite identification.

### 2.4. Data analysis

The raw mass spectrometry data were converted to mzXML format using ProteoWizard. XCMS, running under the R software platform, was used for feature detection, retention time correction, and peak alignment. SIMCA software was used for multivariate statistical analysis. Principal component analysis (PCA) was performed for dimensionality reduction and visualization of global sample distribution. Orthogonal partial least squares discriminant analysis (OPLS-DA) was then used to construct a classification model. Metabolites with a variable importance in projection (VIP) score >1 and *p* < 0.05 (Student´s t-test) were considered significantly different. Identified differential metabolites were annotated using the Fiehn metabolomics database based on retention time and mass-to-charge ratio (m/z).

## 3. Results and discussion

### 3.1. Metabolic profiling and comparative analysis of mung bean varieties

**Fig 1:**
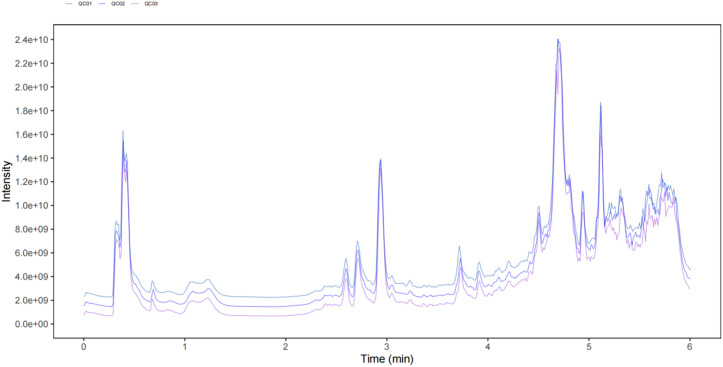
Total ion chromatograms (TICs) of QC samples in positive ion mode, obtained from pooled extracts of all six mung bean varieties. The high consistency in retention time and intensity across samples indicates excellent instrument stability and reproducibility of the analytical method.

**Fig 2:**
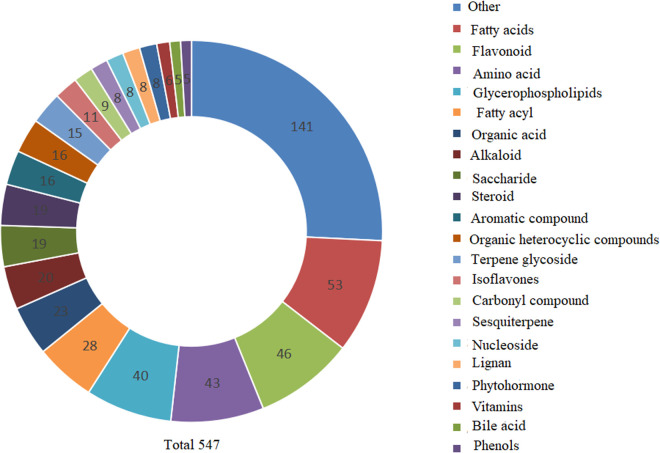
Circular diagram showing the classification and distribution of 547 identified metabolites in six mung bean varieties. The metabolites span diverse categories, with the “Other” group being the most abundant (141), followed by fatty acids (53), phenolic acids (43), amino acids (40), and organic acids (28), illustrating the rich chemical diversity of mung beans.

In order to preliminarily analyze the metabolomic characteristics of six mung bean varieties, their metabolites were analyzed using UPLC-Q-Orbitrap HRMS in positive ion mode. As shown in Fig 1, the total ion chromatograms (TICs) of QC samples (mixed from all six mung bean varieties) exhibited high consistency in both retention times and response intensities across all chromatographic peaks. This high level of overlap indicates minimal instrumental deviation and excellent stability of the data acquisition process. A total of thousands of compound ions were detected across the six mung bean samples. Subsequent compound identification was performed using public databases such as mzCloud and OTCML, as well as an in-house library. A total of 547 metabolites were identified (Fig 2). Among them, the largest proportion belonged to the “others” category (25.78%), followed by fatty acids (9.69%), phenolic acids (7.86%), amino acids and their derivatives (5.12%), organic acids (4.20%), and alkaloids (3.66%). In addition, other compound classes such as sugars, steroids, and glycosides were also detected, reflecting the diverse chemical makeup and rich nutritional composition of mung beans.

**Fig 3:**
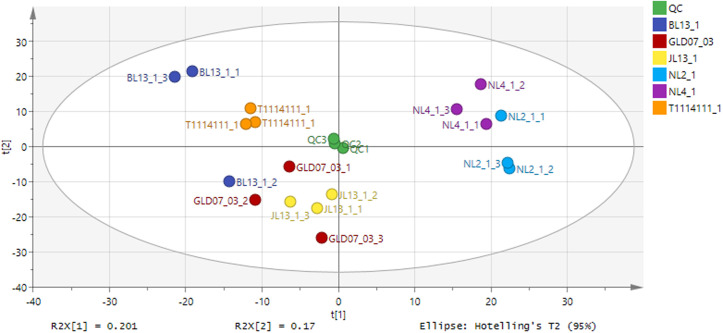
Principal component analysis (PCA) score plot based on the metabolite profiles of six mung bean varieties. Each colored point represents a biological replicate (n = 3) for each mung bean variety: JL13−1 (red), GLD07−03 (dark red), T1114111-1 (orange), BL13−1 (blue), NL2−1 (sky blue), NL4−1 (purple), and QC samples (green). The first two principal components, PC1 (R2X[1] = 20.1%) and PC2 (R2X[2] = 17.0%), together explain 37.1% of the total variance in the dataset. The clear separation among sample groups indicates distinct metabolomic profiles across different mung bean varieties. QC samples cluster tightly, confirming the analytical method’s stability and reproducibility. The Hotelling’s T2 95% confidence ellipse is shown to highlight the variation range within normal biological replicates.

In addition, principal component analysis (PCA) was performed to further compare the differences in metabolite composition among the six mung bean varieties (Fig 3). The analysis results showed that there were significant differences in metabolite composition among different mung bean varieties, and the first principal component (t[1]) explained 20.1% of the characteristics of the dataset, which could effectively distinguish the metabolite characteristics of various varieties. The second principal component (t[2]) explained 17% of the features, further demonstrating the differences in metabolic characteristics between samples. The QC samples were closely clustered in the PCA diagram, which further verified the reliability of the data and the repeatability of the experiment. The overall analysis results showed that different mung bean varieties had significant metabolome differences, which provided a scientific basis for identifying their unique metabolites and nutrients, and laid a foundation for further research.

In this study, the metabolite differences among six mung bean varieties were investigated by various metabolomics analysis methods, and the significant differences in metabolic composition between GLD07_03 and other varieties were revealed, which provided a scientific basis for the metabolic characteristics of different varieties.

**Fig 4:**
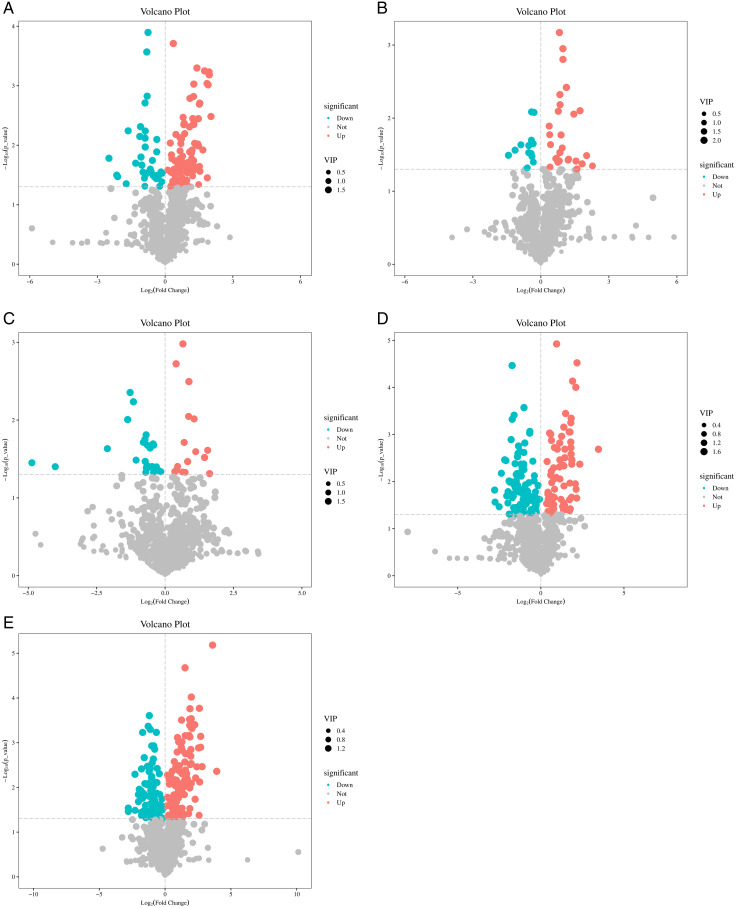
Volcano plots showing differentially accumulated metabolites (DAMs) between GLD07_03 and five other mung bean varieties. Each point represents a single metabolite, with the x-axis showing log2 fold change (FC) and the y-axis showing –log10(*p*-value). Metabolites with VIP > 1 and *p* < 0.05 were considered significantly different. Red and blue dots indicate significantly upregulated and downregulated metabolites, respectively, in GLD07_03 compared with each control group; grey dots indicate non-significant metabolites. A: GLD07_03 vs. T1114111_1; B: JL13_1 vs. GLD07_03; C: GLD07_03 vs. BL13_1; D: GLD07_03 vs. NL2_1; E: GLD07_03 vs. NL4_1.

The volcanic map in Fig 4 analyzes the metabolite differences between different mung bean varieties, revealing significant differences in metabolite composition between GLD07_03 and other varieties. The number of significantly different metabolites and VIP values in each group were different, showing the metabolic characteristics of different varieties. In particular, GLD07_03 showed more up-regulated metabolites in comparison with T1114111_1, JL13_1 and NL2_1, indicating that GLD07_03 had advantages in specific components, while the difference between GLD07_03 and BL13_1 and NL4_1 was small. In general, these differential metabolites can be used as characteristic markers of mung bean varieties, and provide scientific basis for variety identification, functional component analysis and nutritional evaluation of mung bean.

**Fig 5:**
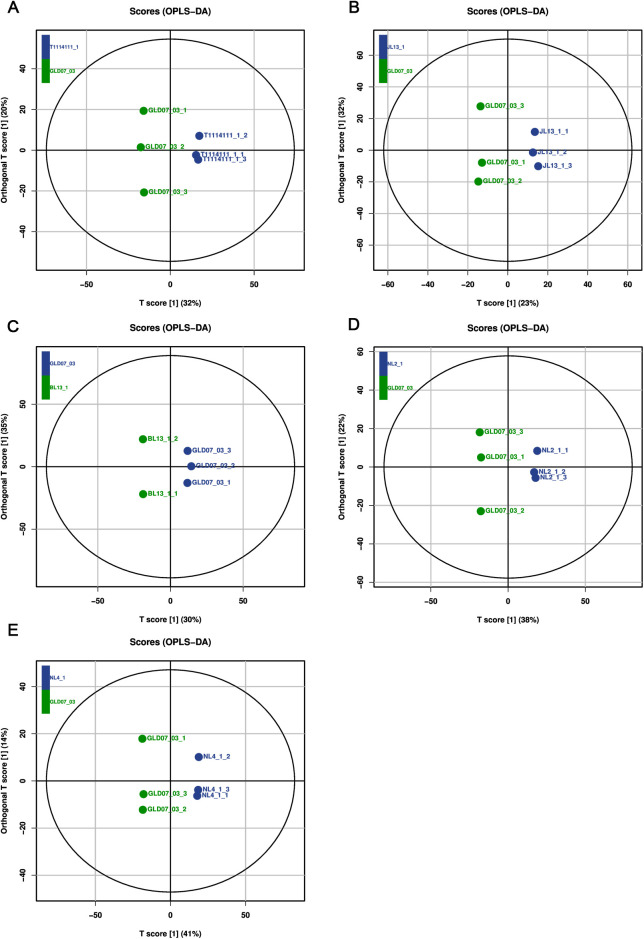
OPLS-DA score plots showing the discrimination of metabolomic profiles between GLD07_03 and other mung bean varieties. Each point represents a biological replicate (n = 3). The X-axis (T score [1]) and Y-axis (orthogonal T score [1]) represent the predictive and orthogonal components, respectively. The numbers in parentheses indicate the explained variance of each component. Clear separation between sample groups suggests significant metabolic differences. A: GLD07_03 vs. T1114111_1; B: JL13_1 vs. GLD07_03; C: GLD07_03 vs. BL13_1; D: GLD07_03 vs. NL2_1; E: GLD07_03 vs. NL4_1.

To optimize the separation between samples, we used OPLS-DA to determine the differences in metabolites across mung bean varieties (Fig 5). OPLS-DA is an extension of the supervised partial least squares regression method, where the feature (X variable) is divided into two parts, and the system variation is divided into two parts, one part models the correlation (prediction) between X and Y, and the other part models the orthogonal (independent of Y) component [22].Therefore, OPLS-DA has the maximum separation of observation classes based on its variables and has better interpretability compared to OPLS-DA. The Q2 value in the model represents the predictive power of the model, and the closer the Q2 value is to 1, the higher the reliability of the model. In the pairwise comparison of different mung bean varieties, the Q2 values of GLD07_03 and JL13_1, GLD07_03 and BL13_1 were relatively high, indicating that these models have strong explanatory and predictive power.

**Fig 6:**
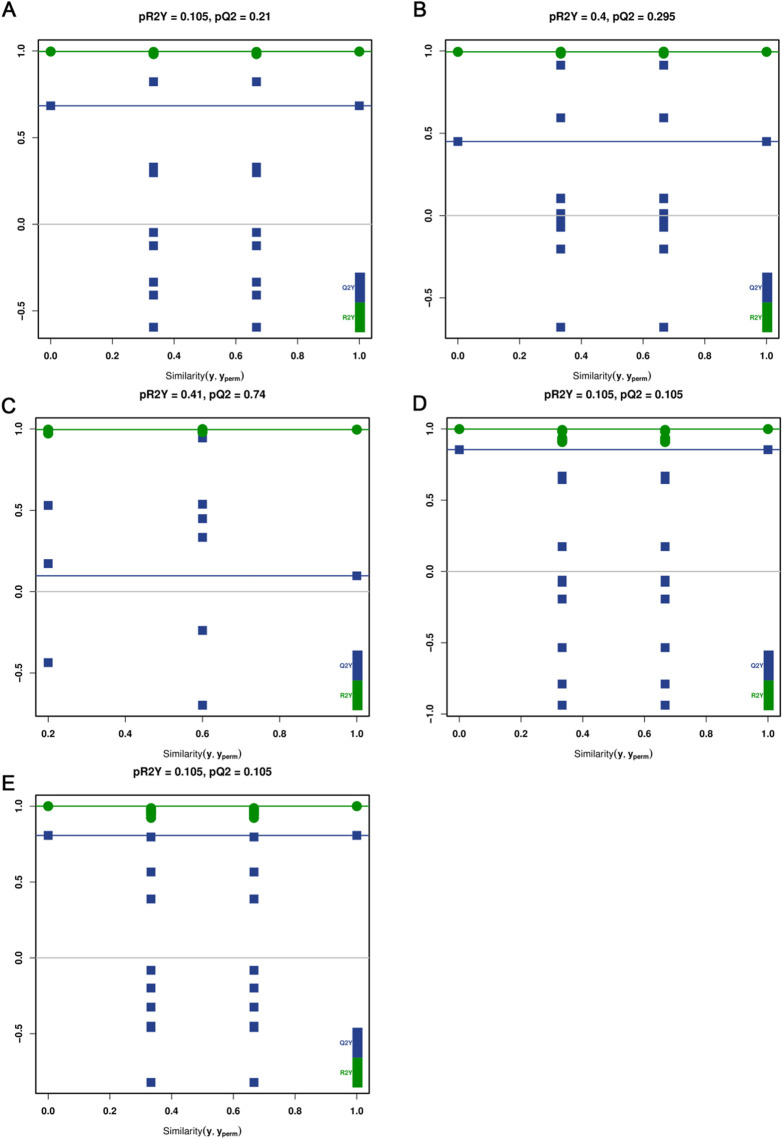
Permutation test plots (n = 200) evaluating the reliability of the OPLS-DA models for pairwise comparisons of GLD07_03 with five other mung bean varieties. The horizontal axis represents the model similarity (R2Y, Q2) obtained after permutation, and the vertical axis shows the corresponding Y-intercepts. The original model values are shown on the right, while the distribution of permuted values is on the left. A valid model is indicated when R2 and Q2 values of the permuted models are lower than those of the original model and the Q2 regression line has a negative intercept. A: GLD07_03 vs. T1114111_1; B: JL13_1 vs. GLD07_03; C: GLD07_03 vs. BL13_1; D: GLD07_03 vs. NL2_1; E: GLD07_03 vs. NL4_1.

Through the substitution test plot (Fig 6), it can be observed that the left blue Q2 points in some groups are lower than the original blue Q2 points in the far right, further proving that these models have high stability and confidence in the specific comparison group.

On this basis, the VIP values of different metabolites were analyzed, and VIP values > 1, |log2 FC| > 1 and *P* < 0.05 were selected. These metabolites showed a significant up-regulated or down-regulated trend in pin-wise comparison of different mung bean varieties. In particular, GLD07_03 was compared with T1114111_1, JL13_1 and NL2_1, which showed that the metabolomic characteristics of GLD07_03 were significantly different from those of these samples, while the differences between GLD07_03 and BL13_1 and NL4_1 were relatively small. This further suggests that differences in metabolite composition between different varieties of mung beans are greater than differences between different samples within the same variety.

In summary, through systematic metabolomic analysis, this study revealed the metabolomic differences among six mung bean varieties, especially GLD07_03 showed significant metabolite differences in comparison with NL4_1, NL2_1 and T1114111_1, indicating that there are significant differences in metabolic characteristics of different mung bean varieties. The results showed that the difference of metabolite composition in different varieties was greater than that in the same variety, which provided important data support for the identification of mung bean varieties, the evaluation of nutrient composition and the study of potential functional characteristics. This study focused on the comparison of samples with significant metabolomic differences, which is helpful to further understand the environmental adaptability and metabolic regulation mechanism of mung bean under different growth conditions, and provide a theoretical basis for the variety improvement and functional development of mung bean.

### 3.2. Analysis of differential metabolites in different mung bean varieties

In this study, the metabolite composition and metabolic pathways of 6 mung bean varieties (GLD07_03, BL13_1, NL2_1, NL4_1, T1114111_1 and JL13_1) were systematically analyzed, and the significant differences in growth adaptability, metabolic activity and stress resistance were revealed. This analysis provides deep insights into the physiological adaptation mechanisms and functional characteristics of mung bean in different environments, and lays a foundation for further research on breeding strategies and functional components.

#### 3.2.1. Metabolites and physiological and metabolic characteristics of BL13_1 mung bean samples.

As shown in Table 1, the BL13_1 sample showed significant advantages in metabolite composition, especially in its energy metabolism and environmental adaptability. It was found that the sample had a high L-lactic acid content, reflecting its high metabolic activity in the glycolytic pathway. This characteristic is closely related to the ability of plants to maintain energy supply in low oxygen or anaerobic environments [23]. The accumulation of L-lactic acid as the end product of glycolysis indicates that BL13_1 can effectively maintain energy metabolism under hypoxic conditions, thus supporting plant growth.In addition, the relatively high content of hydroxymeglutarate in the BL13_1 sample further reveals its high metabolic activity in the Mevalonate pathway. This pathway is a key pathway for the synthesis of membrane lipids and secondary metabolites, and is closely related to membrane stability and the generation of signal molecules [24]. This metabolic characteristic suggests that BL13_1 has significant advantages in maintaining cell membrane structure and promoting signal transduction.

**Table 1:** Relative quantitative comparison of key differential metabolites between GLD07_03 and five mung bean varieties.

Differential metabolites		Relative quantitative value					
Name	Types	GLD07_03	BL13_1	NL2_1	NL4_1	T1114111_1	JL13_1
(7Z,10Z)-7, 10-hexadecadienoic acid	Fatty acids	0.015169729					0.020456682
(R)-(-) -citramalic acid	Fatty acids	0.301656754			0.402898701		
(R) −3-hydroxytetradecanoic acid	Fatty acids	0.044708394		0.018765414	0.025455535		
1, 3-acetone dicarboxyl4-acetylaminobutyric acid	other	0.171255416		0.13424958			
13, 14-dihydroprostaglandin E1	Fatty acids	0.014448402			0.027284118	0.02281434	
2-acetyl-1-alkyl-SN-glycerol-3-phosphate choline	glycerophospholipids	0.722619592		0.353330052	0.345022882		
2, 6-dimethoxy-4-propyl phenol	phenols	0.06883397		0.03637621	0.044235013		
3’,4’,5,7, 8-pentamethoxy-flavone	flavonoid	0.021423962			0.066767882	0.039763333	
3-(3,4, 5-trimethoxyphenyl) −2-allyl-1-alcohol	lignan	0.159784327	0.099289534	0.069679206	0.079289475		0.086156842
3-hydroxysebacic acid	Organic acid	0.006841107			0.011373797		
3-phenylbutyric acid	Phenylpropanoids	0.013222924		0.043848069	0.086099845	0.049496982	
3, 4-dimethoxyphenylacetic acid	Aromatic compound	0.088994447			0.157091334	0.152571718	
4-acetylaminobutyric acid	Amino acid	0.013101359		0.005994399	0.00913178		
4-hydroxybenzoic acid	Aromatic compound	0.107792372				0.191109433	
4-hydroxycinnamic acid	lignan	0.093844355	0.03637393	0.040366801	0.050604549	0.050862911	0.053251354
7-methylguanosine	Nucleotide derivative	0.018692424			0.031251234		
7-methylxanthine	vitamins	0.402938687			0.291485429		
7, 12-dimethylbenzo [a] anthracene	phenanthrene	0.004440436		0.001804602			
Acetic acid -(4-phenyl) −2-butyl ester	other	0.271484666		0.073855568	0.082840942		
Adenosine 3 ‘-phosphate	other	0.062168561		0.206653958	0.214997706		
Adenosine diphosphate	nucleoside	0.00678639	0.005893455	0.009324623			
allantoin	alkaloid	0.48649757			0.555909695	0.598750002	
alpha-D-glucose	vitamins	0.344482707			0.392417832		
Amber semialdehyde	Fatty acids	0.207381955			0.308709333		
Aspartame	Amino acid	0.052745613		0.033320977	0.030549798		
Astilbin	flavonoid	3.046102656		10.63311995	11.09236798		
aztreonam	other	0.002280499			0.008353007		0.003302083
Benazeprilat	Amino acid	0.026303795				0.054856481	
Beta-hydroxyisovaleric acid	Fatty acids	0.139596319			0.150796153		
Chickpea dentin A	isoflavones	0.017652037			0.00627001		0.005327053
Cis-hexadienedionic acid	Fatty acids	0.503808743			0.449837485		
citretin	flavonoid	0.014354003		0.052116032	0.068509816		
coniferin	lignan	0.017536265		0.029902328	0.027025797	0.028409504	
Crocetin	saccharide	0.899968422		0.2391637	0.230836025	0.428436778	
D-alanine	Amino acid	0.074227508		0.111886509	0.09307459		
D-proline	Amino acid	0.267405193			0.332522212		0.338274769
demethoxybetaine	flavonoid	0.128178494		0.445958161	0.488582698		
Deoxycortisone	steroid	0.00508332	0.013808609			0.012260286	
Diacetone alcohol	Carbonyl compound	0.025603503				0.027738583	
Dibutyl phthalate	Aromatic compound	0.007750326		0.006171558			
dihydrocoumarin	Coumarins	0.034609463		0.024776988			
Diisobutyl phthalate	other	0.002931008			0.008059678	0.005920752	
Dinoprostol	phytohormone	0.038637666	0.083900782				
Diphenyl disulfide	other	0.009197619		0.025820041	0.025386035		
DTDP-D-glucose	Nucleotide derivative	0.002458273			0.008683739		
Enrofloxacin	other	0.00366024		0.005654787		0.004600073	
Epicatechin	flavonoid	0.812392914			1.310249319	1.268772502	
Ethyl phosphate	other	0.013714034			0.039215429	0.025789241	
Gallic acid	Aromatic compound	0.005991524				0.021867362	
Gamma-caprolactone	lactones	0.02024481					0.015694406
Gastrin tetrapeptide	Amino acid	0.008379989		0.001952548	0.004163943		
genistein	isoflavones	0.210451618		0.327799399	0.381505252		
geranin	flavonoid	0.056995483				0.091138147	
Ginkgolactone	Terpenolactones	0.009484865				0.022541902	
Glyceric acid	saccharide	0.021786475				0.030766761	
Glycerophosphoryl choline	glycerophospholipids	0.262614827		0.626708542	0.624713861		
glycyrrhizin	Fatty acyl	0.016324228					0.02467552
guanosine	phytohormone	0.020034591		0.032818428	0.034051994		
hexahydropyridine	alkaloid	0.356054942			0.512333956		
Hydroxylsafflower yellow pigment A	lignan	0.002181078		0.007325498			
Hydroxymethylglutaric acid	Fatty acids	0.101787269	0.13615976		0.152290466		
Jasmonic acid	Fatty acyl	0.327911255		0.167332431	0.170536314	0.193295532	
kaempferol	flavonoid	0.018676613			0.030122216	0.041883827	
L-arginine	Amino acid	1.341044327		0.662814454			
L-asparagine	Amino acid	0.045559608	0.058176824			0.059485656	0.060803619
L-aspartate	Amino acid	0.210518486		0.548672043	0.382162929		
L-histidine	Amino acid	0.0579848				0.14270215	
L-kynurenine	Organic oxygen compound	0.021010978				0.060163784	
L-lactic acid	Organic acid	0.108948839	0.171184103				
L-malic acid	Organic acid	2.46780795			5.972726301	4.22040799	
L-serine	Amino acid	0.012614721		0.053123427	0.067121491		
Maleic acid	vitamins	0.658660457			1.544034611	1.107942466	
Malonic acid	Organic acid	0.122374581				0.180468203	
maltohexose	saccharide	0.035738419		0.014943479	0.018155282		
mannitol	saccharide	0.285401975		0.121701728	0.150185161		
Methyl acrylate	Organic acid	0.027737371		0.010291405	0.014203862		
Methyl salicylate	phytohormone	0.051458026	0.011917754	0.007626548	0.007397189	0.011950819	0.0126794
mycoerythritose	other	0.092677427		0.069818773	0.078410687	0.083573306	
myricitrin	flavonoid	0.009511413			0.036876424		
Naringin	flavonoid	0.077645045			0.038420412		
Nicotinamide adenine dinucleotide	nucleoside	0.004386456		0.00773108			
Osmundactone	Organic heterocyclic compounds	0.018126173		0.014230799			
p-hydroxybenzaldehyde	Carbonyl compound	0.480648071		0.725283189	0.780060252	0.646942672	
palmitamide	Organic acid	0.016675525				0.020027822	
panose	saccharide	0.020842839		0.013227557	0.012492606		
Phenylacetic acid	Aromatic compound	0.035748746			0.052409781		
porphyrin	flavonoid	0.036461246				0.057939167	
Propanedioic acid	Organic acid	0.020891184				0.010191248	
Prostaglandin D2	Fatty acids	0.01489195	0.02721055			0.022119951	
puerarin	other	0.080938968				0.226018995	
Quercetin 3-oxyglucoside	flavonoid	0.038895914			0.133742276		
quercitrin	flavonoid	0.008875763		0.015644044			
rutin	flavonoid	0.089760836			0.202272715		
Salviolide	Terpenolactones	0.264930482	0.160123569				
sesamol	Organic heterocyclic compounds	0.28445822		0.135998094	0.134590796		
Simonde lignin	saccharide	0.435997255			0.765307533	0.740057317	
spirovitexin	Terpene glycoside	0.02301418					0.013500406
Spirovitexin	Terpene glycoside	0.02301418	0.013830128				
Stevia A	other	0.105833809			0.144128767		
Succinic acid	Organic acid	0.166246555			0.397066427	0.274697276	
thymoquinone	Carbonyl compound	0.034667502		0.027274531	0.05013776	0.096153426	
Triglyceride triacetate	glycolides	0.014413625		0.012845238			
trigonelline	alkaloid	7.769291356				9.728685898	
trilobin	flavonoid	0.214268594			0.390770494		
Twenty-one carbonic acid	Fatty acids	0.012949829				0.019483633	
Uridine 5’ -diphosphate	nucleoside	0.011454469		0.0293797	0.024483483		
Uridine 5’ -monophosphate	nucleoside	0.031947523		0.137517498	0.119679942		
vanillonamide	Aromatic compound	0.045271631				0.096871239	
xanthine	alkaloid	0.018524994		0.069591068	0.059483364		
xanthoxylin	Carbonyl compound	0.030890056			0.069991191	0.069621632	
β-hydroxyisovaleric acid	Fatty acids	0.139596319		0.13236558			

In terms of plant hormone metabolism, the high levels of dinoprostol and deoxycortisone in BL13_1 samples suggest their potential advantages in growth and development and regulation of environmental responses. Plant hormones play an important role in regulating plant growth, differentiation and coping with environmental stress [25]. Increased levels of these hormones can promote growth and development of plants and improve their resilience in the face of biological and abiotic stresses. In addition, the up-regulation of L-asparagine content further indicates the activity of BL13_1 in the pathway of nitrogen metabolism and protein synthesis. Nitrogen metabolism plays a crucial role in plant growth, amino acid synthesis and overall protein construction [26]. This metabolic characteristic enables BL13_1 to maintain its growth advantage under the condition of nutrient scarcity, indicating that BL13_1 has an efficient metabolic regulation ability in a limited resource environment.

In summary, BL13_1 showed excellent metabolic characteristics in energy metabolism, lipid synthesis and environmental adaptability. The combination of its key metabolic components, such as L-lactic acid, hydroxymethylglutaric acid, dinoprost, deoxycortisone and L-asparagine, allows it to show good adaptability in a variable and challenging growing environment.

#### 3.2.2. Metabolites and physiological metabolic characteristics of NL2_1 mung bean samples.

NL2_1 samples showed significant advantages in the accumulation of nucleosides, amino acids, and flavonoid metabolites (Table 1). Increased levels of nucleosides (such as nicotinamide adenine dinucleotide and uridine 5’ -diphosphate) indicate that NL2_1 is active in energy production and metabolic regulation. These nucleosides are important cofactors in energy metabolism in cells and are involved in a variety of metabolic pathways, such as REDOX reactions and nucleotide biosynthesis [27].At the same time, the upregulation of amino acids (such as L-aspartic acid, D-alanine, L-serine) indicates that NL2_1 has high metabolic activity in nitrogen metabolism and protein synthesis. Amino acids are not only the basic building blocks of proteins, but also play an important role in cell signaling and metabolic regulation [28]. This metabolic characteristic enables NL2_1 to support rapid growth and efficient use of nitrogen sources in resource-limited conditions, thereby improving its environmental adaptability. The up-regulation of flavonoids such as quercetin, genistein and astilbin further enhanced the antioxidant capacity of NL2_1. These compounds reduce cell damage by clearing reactive oxygen species (ROS), thereby improving their survival in stressed environments [29].Flavonoids are an important part of the plant antioxidant defense system, which can effectively protect cells from oxidative stress and maintain the stability of cell function. In addition, the high content of glycerophospholipids (such as glycerophosphoryl choline) suggests that NL2_1 has advantages in maintaining membrane stability and signaling. Glycerophospholipids are an important part of cell membranes, which participate in membrane fluidity and signal molecule synthesis, and help plants maintain physiological homeostasis in complex environments [30]. This glycerophospholipid rich property enables NL2_1 to maintain membrane integrity and functional stability under environmental changes, thereby enhancing its adaptability under stress conditions.

The metabolic characteristics of NL2_1 in nucleoside, amino acid and flavonoid metabolites, combined with its advantages in glycerophospholipid synthesis, make it show outstanding physiological characteristics in terms of rapid growth and environmental adaptation. This metabolic combination not only enhances its antioxidant capacity, but also supports its survival and development in variable environments by improving energy production and maintaining cell membrane homeostasis.

#### 3.2.3. Metabolites and physiological and metabolic characteristics of NL4_1 mung bean samples.

As shown in Table 1, NL4_1 samples showed significant advantages in the accumulation of organic acids, amino acids and flavonoid metabolites. The abundance of organic acids (such as succinic acid and L-malic acid) indicates that NL4_1 samples have a high activity in the TCA cycle. TCA cycle is the core pathway of plant respiration and energy generation, and high levels of succinic acid and L-malic acid indicate that NL4_1 has significant energy metabolism ability in a growing environment with high energy demand [31]. Increased levels of amino acids (such as L-aspartate and D-alanine) further support their metabolic activity in nitrogen metabolism and protein synthesis [28]. By increasing the content of these amino acids, NL4_1 sample enhanced its adaptability and growth ability under high metabolic requirements. The accumulation of flavonoids, such as epicatechin and astilbin, provides NL4_1 with potent antioxidant capacity. Flavonoids act as natural antioxidants in plants, clearing ROS, reducing oxidative damage of cells, and protecting cell structure and function [29]. NL4_1 shows significant advantages in antioxidant defense mechanisms by increasing the concentration of these compounds, thereby improving its survival and stability in stressed environments.

NL4_1 samples demonstrated advantages in energy production and antioxidant defense by enhancing the accumulation of organic acids, amino acids and flavonoid metabolites. The high activity of the TCA cycle and glycolytic pathway provides it with sufficient energy to support rapid growth and cope with environmental stress. In addition, the upregulation of flavonoid metabolites enhances its antioxidant defenses, making it more adaptable to high metabolic demands in complex environments. These metabolic characteristics provide potential application value for NL4_1 in future breeding and functional component research.

#### 3.2.4. Metabolites and physiological and metabolic characteristics of T1114111_1 mung bean samples.

In this study, the metabolic characteristics of mung bean sample T1114111_1 were analyzed in detail, and it was found that the contents of fatty acids, amino acids, organic acids and flavonoid metabolites in the sample were significantly increased (Table 1). This up-regulation of metabolite content is closely related to its advantages in energy production, cell stability, and antioxidant defense.

First, the high levels of L-malic acid, succinic acid and malonic acid detected in the samples indicate that they participate in the active metabolic process of the TCA cycle, which provides stable energy support for the rapid growth and adaptability of plants [31]. As the core pathway of cell metabolism, the tricarboxylic acid cycle meets the requirements of cell biosynthesis and energy requirements by providing ATP and intermediate metabolites [32]. Second, increased levels of flavonoids (such as epicatechin, geranin, and kaempferol) and aromatic compounds (such as gallic acid and 4-hydroxybenzoic acid) in the T1114111_1 sample helped to play a key role in antioxidant defense by clearing excess ROS, thereby reducing the damage caused by cellular oxidative stress. Maintain normal cell function [29].Some studies have pointed out that the antioxidant properties of flavonoids can not only enhance the physiological defense ability of plants, but also have a potential protective effect in response to environmental stress [33]. In addition, the high content of fatty acids and glycerophospholipid metabolites in the samples further enhanced the stability and signaling ability of the cell membrane. These metabolites play a particularly important role in biological and abiotic stress, maintaining the integrity of cell structure and regulating transmembrane signaling to promote adaptive responses [34]. Such enhanced stability and signal transduction provide plants with stronger tolerance and resilience in the face of abiotic stresses such as drought and high temperature [35]. Finally, the high content of alkaloid metabolites in the T1114111_1 sample, especially trigonelline, provides additional antimicrobial and anti-inflammatory defense functions. This alkaloid further improves the overall stress resistance of plants by inhibiting the growth of pathogenic microorganisms and reducing inflammatory responses [36]. The antibacterial properties of trigonelline in various plants have been extensively studied, and its potential in plant disease prevention and control cannot be ignored [37].

In summary, the comprehensive action of various metabolites in the sample of T1114111_1 mung bean makes it show significant advantages in energy metabolism, cell stability and antioxidant defense. This provides a solid metabolic basis for mung bean’s adaptability under different stress conditions, and provides a valuable research direction for improving plant stress resistance through metabolite regulation in the future.

#### 3.2.5. Metabolites and physiological and metabolic characteristics of JL13_1 mung bean samples.

Analysis of JL13_1 mung bean samples revealed its metabolic characteristics, in particular the abundance of amino acids (such as D-proline, L-asparagine) and fatty acid metabolites (such as (7Z,10Z)-7, 10-hexadecadienoic acid) (Table 1). The high content of these metabolites indicates that the sample has significant advantages in nitrogen metabolism, osmotic regulation and membrane stability [28, 34].

As an osmoprotective agent, proline plays a key role in plant response to abiotic stresses such as drought and salt stress. It stabilizes protein structure, protects cells from dehydration damage, and helps maintain cellular water balance [38]. The content of D-proline in JL13_1 samples is prominent, which indicates that JL13_1 has strong adaptability in osmoregulation, which helps plants maintain growth activity in extreme environment. L-asparagine is another amino acid that is significantly present in JL13_1 samples and plays an important role in nitrogen storage and transport. Under the condition of limited nitrogen in plants, asparagine can promote nitrogen redistribution, thereby maintaining metabolic activities and growth [39]. This characteristic enables JL13_1 sample to maintain a stable physiological and metabolic state under the condition of nutrient limitation. The presence of fatty acids such as (7Z,10Z)-7, 10-hexadecadienoic acid in JL13_1 samples suggests that it has advantages in the construction and stability of cell membranes. Fatty acids play a key role in maintaining membrane structural integrity and fluidity, which is critical for responding to temperature fluctuations and changes in osmotic pressure [34]. The abundance of fatty acid metabolites helps to improve the adaptability of cell membranes and ensure that cells maintain homeostasis in complex environments.

Overall, the metabolic characteristics of JL13_1 samples reflect the physiological advantages of JL13_1 in coping with environmental stress. The high content of amino acids and fatty acids supports its advantages in osmoregulation, nitrogen metabolism and membrane stability, making it potentially useful in plant breeding.

#### 3.2.6. Metabolites and physiological metabolic characteristics of GLD07_03 mung bean samples.

GLD07_03 Mung bean samples showed significant advantages in defense and stress resistance metabolites, and its metabolic characteristics were mainly reflected in the enrichment of defense-related metabolites such as lignans (such as 3-(3,4,5-trimethoxyphenyl)-2-allyl-1-ol, 4-hydroxycinnamic acid, terpenoid lactones (such as salicylate) and methyl salicylate (Table 1). These compounds are produced mainly through the phenylalanine pathway, giving GLD07_03 sample powerful antibacterial and antioxidant functions, contributing to its self-protection ability under pathogen invasion and environmental stress [40].

Lignans play an important role in plant defense, and their antibacterial and antioxidant properties have been confirmed in various studies [41]. GLD07_03 samples rich in 3-(3,4, 5-trimethoxyphenyl) −2-allyl-1-ol and 4-hydroxycinnamic acid can not only directly inhibit the growth of pathogens, but also reduce the accumulation of ROS under stress conditions by inducing antioxidant enzyme activity, and protect cells from oxidative damage. Terpenolactones such as salviolactone also play an important role in plant response to pests and abiotic stresses. They can improve the defense capability of plants by inhibiting the biosynthetic pathway of pathogens and promoting the strengthening of cell walls [42]. As a key signaling molecule, methyl salicylate plays a central role in activating systemic acquired resistance (SAR) [43]. SAR is a whole-plant immune mechanism that can enhance plant resistance to broad-spectrum pathogens through signal transduction pathways [44]. The high content of methyl salicylate in GLD07_03 samples further proves that GLD07_03 has strong signal transmission and defense response ability. The high content of carbohydrate and nucleoside metabolites in GLD07_03 samples, such as colocin, maltohexose and adenosine diphosphate, provided energy reserve and osmoregulation functions under stress. Carbohydrate metabolites not only play an important role in the regulation of cell osmosis, but also serve as carbon sources to support physiological activities of plants under stress conditions [45]. As a nucleoside metabolite, adenosine diphosphate is involved in energy metabolism and signal transmission, supporting plants to maintain metabolic activity under adverse conditions [46].

Overall, GLD07_03 mung bean samples showed significant physiological adaptability to pests and environmental stresses through accumulation of a range of defense and resistance metabolites. This not only revealed the ecological adaptation strategy of GLD07_03, but also provided a scientific basis and application prospect for the study of stress resistance and the development of functional components of mung bean.

## 4. Conclusions

In this study, the metabolite composition and metabolic differences of six mung bean varieties (GLD07_03, BL13_1, NL2_1, NL4_1, T1114111_1 and JL13_1) were systematically revealed by non-targeted metabolomics analysis. The results showed that there were significant differences in growth adaptability, metabolic activity and stress resistance among various varieties, which provided scientific basis for understanding the physiological adaptation mechanism of mung bean. BL13_1 showed high metabolic activity in glycolysis and mevalonate pathways, which enhanced its energy metabolism and cell membrane stability under hypoxic conditions. Its high levels of plant hormones and nitrogen metabolic activity support its growth advantage when nutrients are scarce. GLD07_03 samples are rich in defensive metabolites such as lignans, terpenoids and methyl salicylate, highlighting their antimicrobial and antioxidant potential. NL2_1 and NL4_1 have advantages in the accumulation of amino acids, nucleosides and flavonoids, reflecting their antioxidant and energy metabolism capabilities. The high content of fatty acids and alkaloids in T1114111_1 samples improved cell stability and antibacterial activity, while the superior content of amino acids and fatty acids in JL13_1 samples contributed to osmotic regulation and cell membrane homeostasis. The overall analysis showed that the metabolism of different mung bean varieties was significantly different, which provided important data support for variety identification, nutritional evaluation and functional development of mung bean, and provided a theoretical basis for future breeding and research.These results fill the gap in the scientific understanding of mung bean varieties on the metabolic level, and lay a foundation for future research on the ecological adaptability, metabolic regulation and functional components of mung bean.
